# Rapid case-based mapping of seasonal malaria transmission risk for strategic elimination planning in Swaziland

**DOI:** 10.1186/1475-2875-12-61

**Published:** 2013-02-11

**Authors:** Justin M Cohen, Sabelo Dlamini, Joseph M Novotny, Deepika Kandula, Simon Kunene, Andrew J Tatem

**Affiliations:** 1 Clinton Health Access Initiative, Boston, MA, USA; 2 National Malaria Control Programme, Mbabane, Swaziland; 3 Global Health Group, University of California, San Francisco, USA; 4 Department of Geography and Environment, University of Southampton, Southampton, UK; 5 Fogarty International Center, NIH, Bethesda, MD, USA

## Abstract

**Background:**

As successful malaria control programmes move towards elimination, they must identify residual transmission foci, target vector control to high-risk areas, focus on both asymptomatic and symptomatic infections, and manage importation risk. High spatial and temporal resolution maps of malaria risk can support all of these activities, but commonly available malaria maps are based on parasite rate, a poor metric for measuring malaria at extremely low prevalence. New approaches are required to provide case-based risk maps to countries seeking to identify remaining hotspots of transmission while managing the risk of transmission from imported cases.

**Methods:**

Household locations and travel histories of confirmed malaria patients during 2011 were recorded through routine surveillance by the Swaziland National Malaria Control Programme for the higher transmission months of January to April and the lower transmission months of May to December. Household locations for patients with no travel history to endemic areas were compared against a random set of background points sampled proportionate to population density with respect to a set of variables related to environment, population density, vector control, and distance to the locations of identified imported cases. Comparisons were made separately for the high and low transmission seasons. The Random Forests regression tree classification approach was used to generate maps predicting the probability of a locally acquired case at 100 m resolution across Swaziland for each season.

**Results:**

Results indicated that case households during the high transmission season tended to be located in areas of lower elevation, closer to bodies of water, in more sparsely populated areas, with lower rainfall and warmer temperatures, and closer to imported cases than random background points (all p < 0.001). Similar differences were evident during the low transmission season. Maps from the fit models suggested better predictive ability during the high season. Both models proved useful at predicting the locations of local cases identified in 2012.

**Conclusions:**

The high-resolution mapping approaches described here can help elimination programmes understand the epidemiology of a disappearing disease. Generating case-based risk maps at high spatial and temporal resolution will allow control programmes to direct interventions proactively according to evidence-based measures of risk and ensure that the impact of limited resources is maximized to achieve and maintain malaria elimination.

## Background

Recent reductions in malaria coupled with increased funding have resulted in a renewed focus on malaria eradication [1]. Many countries have adopted a goal of national malaria elimination [2], and the World Health Organization (WHO) recognizes 17 as having pre-elimination or elimination programmes [3]. Achieving elimination is an operationally challenging endeavour requiring a strong evidence base and targeted interventions [4].

Many of the operational requirements for malaria elimination, including identifying residual transmission foci [5], targeting vector control and case detection to high risk areas [6], focusing not only on clinical disease but also asymptomatic infections [4], and managing importation risk [7] can be facilitated by accurate and timely creation of malaria risk maps. For example, risk maps may allow proactive deployment of vector control measures to high-risk areas to prevent local transmission, or suggest areas where active case detection may be used to identify and treat remaining parasite reservoirs.

Parasite rate-based maps for malaria are now widely available [8-11], but infection prevalence is a poor metric for measuring malaria at very low levels of endemicity due to the enormous surveys required for precise measurement in such contexts [12]. Additionally, while the Bayesian approaches typically used provide valuable information on spatial uncertainty [13], they can require substantial resources and computing time to produce, neither of which may be aligned with the needs of an elimination programme. In very low transmission environments, diagnostically confirmed malaria incidence provides a more useful measure than prevalence. Understanding the epidemiology of these confirmed cases requires differentiating between imported and locally acquired cases [14]. As endemic transmission declines, an increasing proportion of incident cases may be attributed to transmission chains traced directly to imported cases [15], and such imported cases may therefore become increasingly important drivers of local transmission.

Swaziland has achieved its lowest ever recorded malaria prevalence in recent years [16], and it aims to achieve elimination by 2015 [17]. WHO certification of elimination requires achieving an absence of all local transmission for three years, as well as a sufficiently strong surveillance system to prove that cases would have been identified had they occurred [18]. In sub-Saharan Africa, only Lesotho and the island of Mauritius are considered malaria-free, and only the latter achieved that status through active measures. Achieving this goal in Swaziland will require identifying and interrupting remaining foci of endemic transmission [5] and preventing onward transmission from the imported cases that will continue to occur from endemic neighbours [4]. Focusing limited resources on hotspots of transmission rather than aiming for untargeted coverage could considerably improve the impact of interventions [6].

Swaziland instituted a reactive surveillance system in 2009 in which all notifiable diseases, including malaria, identified in health facilities are reported to a central toll-free hotline. Entry of any malaria case into the central database triggers an automated phone short message service (SMS) to the national malaria control programme (NMCP) with basic details on the patient. Surveillance agents then obtain the case patient’s contact details and directions to his or her household from the health facility where the diagnosis was made. The patient is interviewed at the household after identification; the protocol from 2009–2011 was to complete this follow up within seven days of the case report, though the programme now attempts to investigate within 48 hours. Among other information, the interview ascertains travel history, to assist with categorizing the infection as locally acquired or imported, and geocoordinates for the house location. If there is suspicion of local transmission, family members and neighbours living within 1 km of the index case are screened for malaria by rapid diagnostic test, and any individuals who test positive are referred to the nearest health facility for treatment.

Such a surveillance system is a crucial component of an elimination strategy, but achieving and maintaining elimination will require complementing it with proactive case detection to seek out cases that may never come into contact with reporting health facilities [4]. In Swaziland’s 2010 Malaria Indicator Survey, 53.5% of women and 67.4% of children were reported as attending a health facility when febrile [16], leaving a substantial fraction of potential infections that may be missed by passive surveillance. Furthermore, molecular diagnostic methods have indicated that in low transmission settings such as Swaziland, a majority of infections may be asymptomatic and thus will not be identified by the passive surveillance system [19]. Understanding and investigating all regions where unobserved transmission may be occurring will be required before elimination can be achieved.

Malaria prevalence in Swaziland is too low for standard parasite rate-based mapping to be useful [16], so individual case-based approaches are required to predict risk across the country. This investigation seeks to generate maps of malaria risk at fine spatial resolution from existing case-based surveillance data, including the locations of imported cases. Transmission risk maps are derived separately for the high and low transmission seasons in case the key determinants of transmission change over the course of the year. Accurate case-based risk mapping of this kind will help Swaziland to target its vector control and surveillance activities most effectively.

## Methods

### Malaria data

**Figure 1:**
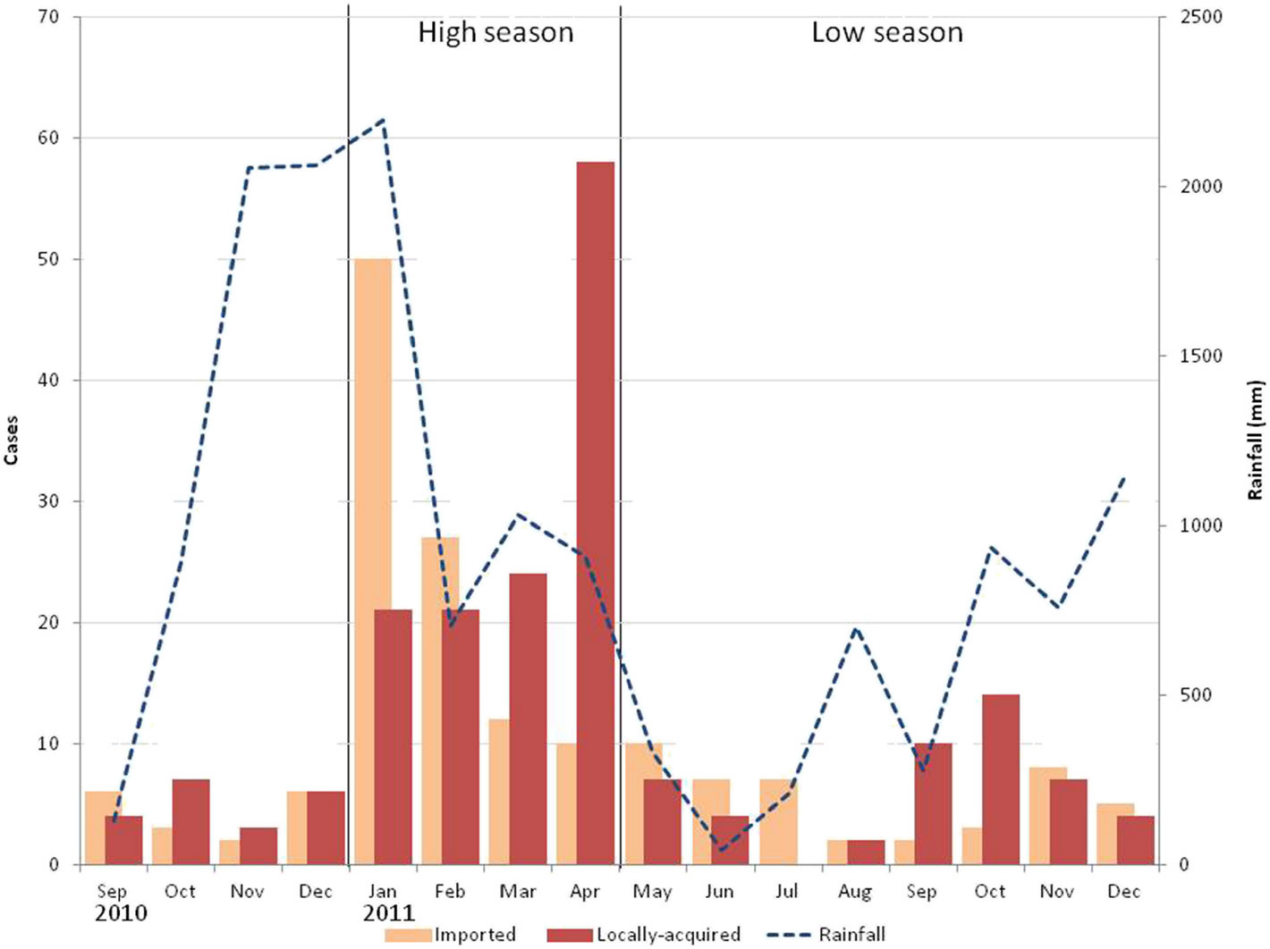
**Cases identified by passive and reactive surveillance in Swaziland after implementation of case investigation and concurrent rainfall.** The high transmission period of January to April 2011 was mapped separately from the low season of May to December. Cases from 2010 were not used for mapping.

This investigation investigated transmission risk in Swaziland based on malaria cases identified during 2011 (Figure 1). Household locations of cases identified by passive or reactive case detection were categorized by the NMCP according to reported travel history. Infected individuals reporting no travel, whether abroad or within Swaziland, in the previous two weeks were assumed to represent locally acquired cases. Infected individuals who reported travel abroad to endemic countries within biologically meaningful windows were assumed to represent imported cases. Those who reported travel to known endemic regions of Swaziland were assumed to have “intraported” infections to their household locations and were grouped together with the imported cases. Cases from 2011 were divided according to whether they occurred during the higher transmission season from January to April or the lower season from May to December. The higher transmission season followed the increase in rains in September as well as an annual peak in malaria importation in January following the end of holiday season travel.

### Predictor variables

Gridded maps of spatial covariates were collated to describe weather, geography, land cover, population density, vector control and imported infections (Table 1). Rainfall and temperature strongly impact the biology of malaria transmission [20], while elevation and topography have been demonstrated to influence risk through their effects on temperature and suitability for mosquito breeding [21]. The topographic wetness index, a measure representing the amount of water that should enter a given spatial unit divided by the rate at which the water should flow out of that unit, was calculated from elevation as a measure for suitability for mosquito breeding habitat [21,22]. Suitability for mosquito habitat was also described using remotely sensed imagery [23]. The normalized difference vegetation index (NDVI) [24] and the modified normalized difference water index (NDWI) [25] were calculated from a single Landsat Enhanced Thematic Mapper (ETM) image from March 2009 with spatial resolution 30 m. Images from the beginning of each season could not be identified due to cloud cover and satellite malfunctions. Densely populated areas may face substantially different malaria risks from very sparsely populated, rural areas [26], and the susceptibility of these populations is influenced by the control measures currently being implemented. The NMCP records the geolocations of all distributed nets but only tallies the number of households receiving indoor residual spraying (IRS) within each of the localities in the country. These IRS data were aggregated to the level of the 55 constituencies since no geographic data on the localities could be identified. Finally, cases classified by the NMCP as imported or intraported based on travel history to endemic areas were also used as predictor variables under the assumption that at the very low prevalence levels observed in Swaziland, infections from other regions play a large role in sparking transmission [27]. Time-varying covariates were generated separately for the high and low seasons where possible as described in Table 1.

**Table 1:** Variables used to analyse transmission risk

**Category**	**Variable**	**Input data**	**Manipulation**	**Predictors for high season**	**Predictors for low season**
Weather	Rainfall	Monthly rainfall from 12 weather stations across the country	Ordinary kriging in R [28] to interpolate values across the country	Mean, minimum, maximum and summed monthly rainfall from November 2010 through February 2011	Mean, minimum, maximum and summed montly rainfall from March 2011 through October 2011
	Temperature	Long-term averages, maximums, and minimums from WorldClim datasets [29]		Long-term average, maximum, and minimum temperature	Long-term average, maximum, and minimum temperature
Geography	Elevation and topography	90 m digital elevation model from the Radar Topography Mission [30,31]	Topographic wetness index [32] calculated from the elevation map with the System for Automated Geoscientific Analyses (SAGA) [33]	Elevation, topographic wetness index	Elevation, topographic wetness index
Land cover	Vegetation	Landsat Enhanced Thematic Mapper (ETM) image from March 2009 with spatial resolution 30 m	Used to calculate the normalized difference vegetation index (NDVI) [24] and the modified normalized difference water index (NDWI) [25], as well as the distance to the grid-cells with highest (99.99^th^ percentile) NDWI	NDVI, NDWI, distance to highest NDWI	NDVI, NDWI, distance to highest NDWI
	Water bodies	Map of water bodies and irrigation zones from the Food and Agriculture Organization of the United Nations's Aquastat website [34], map of rivers from Diva GIS [35]	Distance to the nearest water body or irrigation zone and distance to nearest river calculated in ArcGIS [36]	Distance to nearest water body, distance to nearest river	Distance to nearest water body, distance to nearest river
Population	Population density	Gridded 100 m resolution population map from the AfriPop project [37,38]		Population density	Population density
Vector control	Indoor residual spraying (IRS)	Date and number of houses covered by IRS in each of the 55 constituencies	Number of households receiving IRS in each region summed for the six months prior to each transmission season	Houses sprayed per population from July to December 2010	Houses sprayed per population from November 2010 to April 2011
	Bed nets	Geolocations of all nets distributed since January 2010	All nets distributed before the start of each transmission summed at a 1 km resolution	Nets distributed per population from January to December 2010	Nets distributed per population from January 2010 to April 2011
Importation	Imported cases	Household locations of all identified imported cases	Distance to the nearest imported case household identified from two months prior to the start of the transmission season to two months prior to the end	Distance to the nearest of 89 imported case households identified from November 2010 to February 2011	Distance to the nearest of 54 imported case households identified from March to October 2011

All variables were resampled to 100 sq m, producing maps with 2.4 million grid cells across the country.

### Transmission risk modelling

Values for each of the covariates in Table 1 were compared between the locations of the households of patients identified with locally acquired infections and population weighted, randomly selected “background” points from across Swaziland. Background points do not necessarily indicate the absence of transmission, but instead characterize the environment of the country [39] in the places where people live. A sample of 10,000 background points [39,40] was selected randomly but proportionately to population density across Swaziland using the Geospatial Modelling Environment v0.6 [41]. The population-based weighting ensured that the territory sampled by the background points was comparable to the locations from which local cases arose [42]. The locations of local case households in the high and low transmission seasons were compared to the locations of the background locations to identify conditions under which local transmission is likely to occur. This comparison was based on the assumption that case household locations were indicative of where transmission occurred. Mean values of each of the predictor variables were compared between case households and the background locations using Satterthwaite t-tests for unequal variance [43].

Given the potential importance of imported infections for sustaining malaria transmission in Swaziland [15], a second analysis investigated risk factors associated with whether or not an imported case led to onward transmission. Each imported infection identified in 2011 was classified as to whether or not a locally acquired infection was identified within a space-time cylinder consistent with onward transmission. Local transmission was assumed if the household of a locally acquired infection was identified within 3 km of the imported case household (based upon the typical maximum dispersal range of African vectors [44]) and three to six weeks after identification of the imported case (assuming transmission would require two to three weeks for parasite development inside the mosquito vector, symptoms would develop in an infected human within the following one to two weeks [45], and up to an additional week might elapse before the case patient appeared in passive surveillance reports).

A logistic regression mixed model predicting whether or not an imported case was associated with a locally acquired case was fit using the GLIMMIX procedure in SAS software, Version 9.2 of the SAS System for Windows [46]. An exponential structure was used to account for spatial autocorrelation [47]. Predictive variables included in this model were the same as above but additionally included time-varying rainfall variables describing total rainfall two, four, six, and eight weeks prior to identification of the imported case. Initial models were fit to identify the variables from each category (weather, geography, land cover, population density, and vector control) most associated with transmission, and then each of those was entered jointly into a final model. Variables were removed in order of least significance until all remaining in the model were significant at α = 0.10.

### Mapping

The regression tree classification approach ‘Random Forest’ [48] was applied using the R [49] package ModelMap [50] to model the probability of a locally acquired case occurring in each 100 sq m location across Swaziland. Regression trees create a series of rules to partition the data into a set of groups that are as homogenous as possible with respect to the outcome [51]. For example, one such rule might differentiate the locations of case households from those of control households based on elevation below a certain threshold, while another rule might further divide the data based on rainfall within specific bounds. In the Random Forest approach, the data are repeatedly split according to many different branching "trees" of this type, and the final prediction is made by averaging across all of the individual trees [48]. The free ModelMap package contains a detailed tutorial and example code for implementing Random Forest in R [52].

To assess the accuracy of model predictions, eighty percent of the locally-acquired cases observed in 2011 were selected at random for training the algorithm, with the other 20% were used for testing. All of the above predictor variables were included in the fitting step to produce a model predicting the probability of a local case occurring at a particular location as a function of the combined covariates. Model quality was assessed by examining calibration plots [53] and the area under the curve (AUC) on receiver operating characteristic (ROC) graphs. The fit model was then applied in conjunction with the 100 m spatial resolution gridded datasets of all included predictive variables to generate a map of predicted risk across Swaziland. Models and maps were generated separately for the high and low seasons.

### Prospective validation

Although the AUC values suggested the ability of the model to predict the locations of local cases not used in the model fitting, these test points were obtained over the same time period as the training points and may thus not be indicative of the true value of the risk map for prospective prediction. To examine the utility of the maps for predicting the occurrence of cases in future transmission seasons, an additional dataset of locally acquired cases identified during 2012 was used for validation of the 2011-based risk map. The predicted risk at the locations of 2012 case households according to the Random Forest maps was compared to predicted risk at other random locations across Swaziland. These "control" locations were selected in two ways: first, 10,000 points were randomly selected from across Swaziland, and second, 10,000 points were selected randomly but proportionally to population density. The high transmission season risk map was used to examine predicted risk at the locations of households of cases occurring from January to April 2012, while the low transmission season risk map was used for subsequent months.

## Results

Of the 372 malaria cases investigated during 2011, 191 (51.3%) were classified as locally acquired and 170 were classified as imported (45.7%). Eleven were either not classified or categorized as cryptic [54] and were excluded from analysis. A total of 314 of these cases had valid coordinates that matched the region of the country in which the case was reported to live on the investigation form and were used in analyses. These cases included 118 locally acquired infections from the higher transmission season of January to April and 44 from the lower transmission season of May to December. There were 152 imported infections during 2011 with valid coordinates, of which 143 (94.1%) originated outside of Swaziland.

Characteristics of the locations of case households and the background points during the two study periods are contrasted in Table 2. Of the 152 imported cases used in analysis, 12 (7.9%) were associated with a locally acquired case occurring within 3 km and after three to six weeks. All 12 originated abroad. The final model predicting the probability of an imported case in 2011 leading to onward transmission is reported in Table 3.

**Table 2:** Characteristics of the locations of case households in the high and low transmission season of 2011 in Swaziland, compared against the characteristics of 10,000 population-weighted random background points

	**High season (Jan to Apr)**					**Low season (May to Dec)**					**Comparison of case locations between seasons**		
	**Cases**	**Background**	***t*-test**	**df**	**p**	**Cases**	**Background**	***t*-test**	**df**	**p**	***t*-test**	**df**	**p**
N	118	10,000				44	10,000						
Mean rainfall (mm)	132.28	150.32	8.82	130	**0.000**	45.32	47.15	1.59	46	0.119	−37.47	168	**0.000**
Summed rainfall (mm)	529.11	601.28	8.82	130	**0.000**	362.58	377.22	1.59	46	0.119	−13.62	118	**0.000**
Min rainfall (mm)	46.80	63.40	14.10	134	**0.000**	7.04	3.45	−6.47	46	**0.000**	−31.11	163	**0.000**
Max rainfall (mm)	182.72	198.60	6.45	130	**0.000**	98.67	103.24	0.74	46	0.466	−12.62	61	**0.000**
Annual mean temperature (°C)	21.57	19.40	−24.04	137	**0.000**	21.42	19.40	−11.42	47	**0.000**	−0.73	70	0.470
Max temperature of warmest month (°C)	30.69	28.06	−21.43	134	**0.000**	30.42	28.06	−10.60	47	**0.000**	−1.07	75	0.288
Min temperature of coldest month (°C)	8.79	7.47	−16.39	133	**0.000**	9.13	7.47	−8.73	47	**0.000**	1.67	63	0.099
Elevation (m)	364.23	678.25	22.13	139	**0.000**	382.09	678.25	10.19	47	**0.000**	0.56	68	0.579
Topographic wetness index	20.34	16.61	−12.89	127	**0.000**	18.54	16.61	−3.62	46	**0.001**	−2.96	74	**0.004**
Landsat NDVI	0.41	0.44	4.26	128	**0.000**	0.42	0.44	1.92	47	0.061	0.56	81	0.580
Landsat NDWI	−0.43	−0.43	0.95	136	0.346	−0.45	−0.43	2.50	47	**0.016**	−1.95	62	0.056
Distance to lake or irrigation	6667	16,860	16.19	140	**0.000**	9298	16,860	4.31	47	**0.000**	1.42	58	0.161
Distance to river	1424	1660	2.82	129	**0.006**	1380	1660	2.03	47	**0.048**	−0.28	81	0.781
Population per 100 m by 100 m cell	1.48	11.64	17.11	597	**0.000**	1.80	11.64	9.05	66	**0.000**	0.30	61	0.764
IRS sprayed houses per person	0.08	0.06	−2.39	125	**0.018**	0.16	0.05	−3.66	47	**0.001**	2.30	63	**0.025**
Nets per person	44.78	14.25	−1.30	123	0.197	10.42	0.11	−2.57	47	**0.013**	−1.44	130	0.152
Distance to imported case (m)	9359	11,306	2.89	128	**0.005**	8827	10,781	2.54	48	**0.014**	−0.52	120	0.602

**Table 3:** Mixed model predicting whether or not an imported case identified in 2011 was found to be associated with a locally acquired case occurring three to six weeks later within 3 km

**Effect**	**Estimate**	**Standard error**	**t Value**	**Pr > |t|**
Intercept	−122.940	37.553	−3.270	0.001
Annual mean temperature	−0.928	0.280	−3.320	0.001
Max temperature of warmest month	0.935	0.257	3.650	0.000
Min temperature of coolest month	0.390	0.089	4.360	<0.001
Elevation	0.024	0.008	2.860	0.005
Low season *vs* high season	−3.043	0.812	−3.750	0.000
NDWI	15.248	6.396	2.380	0.019
Distance to river	0.001	0.000	3.230	0.002
IRS houses per person	−3.409	1.744	−1.960	0.053

**Figure 2:**
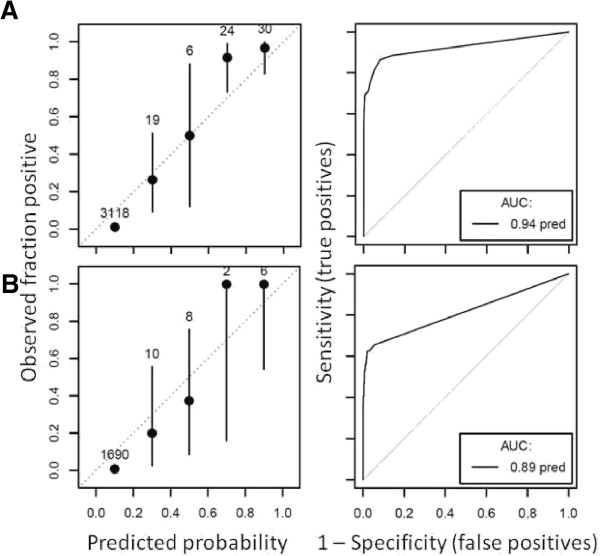
**Calibration (left) and receiver-operator characteristic (ROC) (right) plots to assess model quality for (A) the high season model and (B) the low season model.** The calibration plot suggests no bias if observed standard errors overlap the diagonal. Area under the curve (AUC) in the ROC plot will be 0.5 if the model is no better than random assignment.

**Figure 3:**
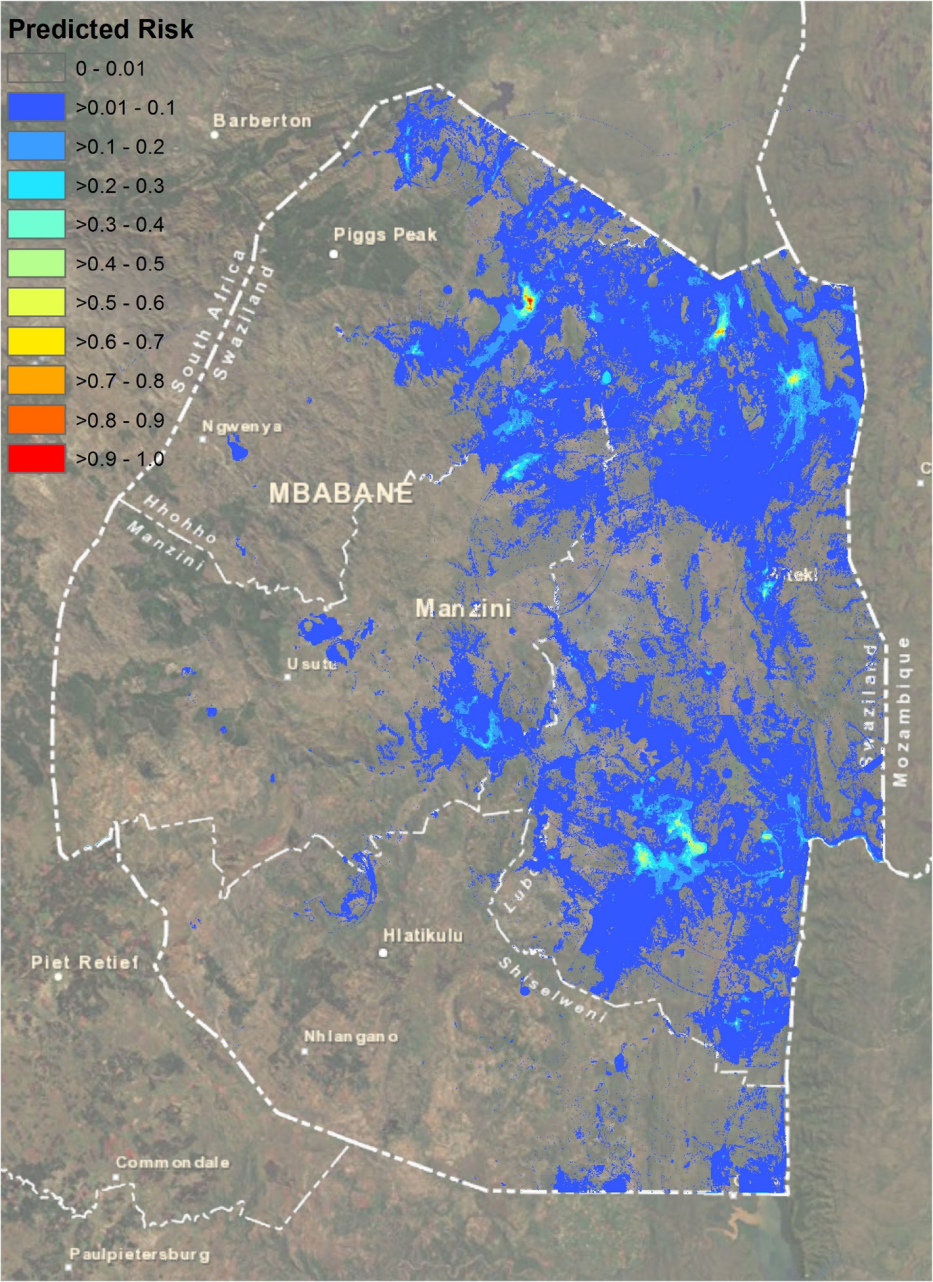
Predicted probability map for presence of locally acquired malaria cases in Swaziland during the high transmission months of January to April 2011.

**Figure 4:**
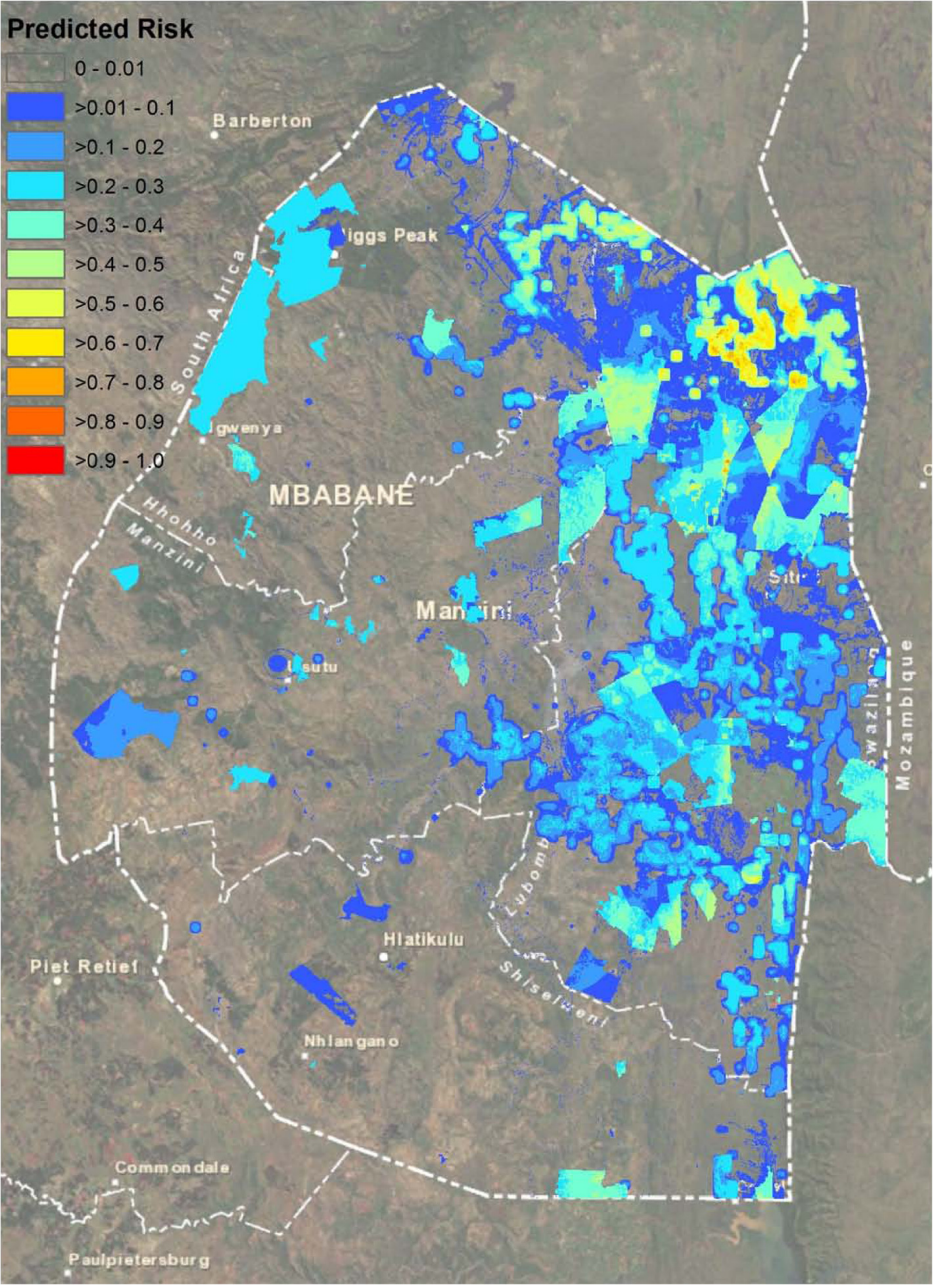
Predicted probability map for presence of locally acquired malaria cases in Swaziland during the low transmission months of May to December 2011.

The ROC plot for the high-season Random Forest model suggested very strong model prediction with AUC = 0.94 (Figure 2A). Judging by the mean decrease in accuracy, model predictions were most dependent upon, in order of descending importance, the distance to the areas with highest NDWI, the distance to the nearest imported case, the distance to lakes, the NDVI, and the TWI. Least important variables were coverage with bed nets, and the mean and sum of rainfall. Model fit was poorer for the low-season model, with AUC = 0.89, likely due at least in part to the smaller sample size (Figure 2B). The model was most dependent on the minimum, mean, summed and maximum rainfall, while least important variables included coverage with bed nets and distance to rivers. Figure 3 and Figure 4 depict the maps generated from the predictive models for the high and low seasons respectively.

**Figure 5:**
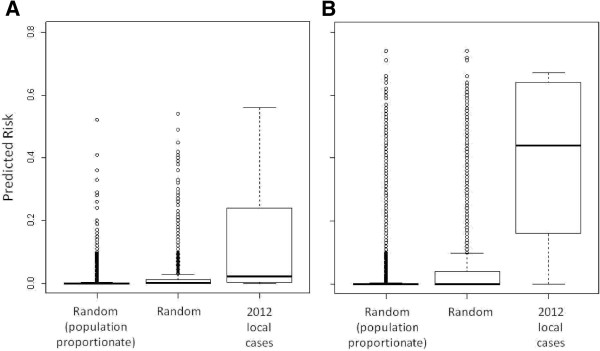
Comparison of predicted risk at 10,000 random locations (sampled first proportionally to population distribution and second at random from across the country) to predicted risk at the location of actual local case households from A) the high transmission season of January-April and B) the low transmission season from May through October.

Twenty-five locally acquired cases were identified by the NMCP from January to April 2012, and 10 from May to October (the most recent data available at the time of analysis). The locations of the 25 high-season cases in 2012 were predicted to have a median risk of 3.4% (interquartile range = 0.4%-12.0%), compared to 0.2% (0.0%-1.2%) for random points from across the country (t = −2.92, p = 0.008) and 0.0% (0.0%-0.2%) for random points sampled proportionately to population density (t = −3.14, p = 0.005) (Figure 5A). The locations of the 10 cases from May to October were predicted to have a median risk of 44.0% (16.8%-64.0%), compared to 0.0% (0.0%-0.2%) for random points from across the country (t = −4.57, p = 0.001) and 0.0% (0.0%-4.8%) for random points sampled proportionately to population density (t = −4.23, p = 0.002) (Figure 5B).

## Conclusions

As countries move towards elimination of malaria, ongoing endemic transmission will become limited to residual foci, and the importance of preventing onward transmission from imported infections will increase [4]. The results of this investigation suggest that both of these epidemiological changes are already well underway in Swaziland. The maps generated here can be applied to target surveillance and vector control to eliminate the remaining foci of transmission in Swaziland and minimize the potential for transmission from imported cases elsewhere. Doing so will improve the efficiency of resource use and have greater impact than aiming for universal coverage everywhere [6].

These risk maps highlight a few areas of Swaziland at very high predicted risk, broad regions at low levels of risk, and many places where risk is estimated to be non-existent. Validation against cases that occurred during 2012 confirm that the areas of predicted risk are the likely locations of future transmission. Accordingly, the areas of highest predicted risk likely represent residual transmission foci where interventions must be targeted to ensure cessation of endemic transmission. The appropriate strategy to minimize the potential for transmission in the low-risk regions identified here will depend upon available resources and Swaziland's risk tolerance. For example, ensuring all areas with any predicted risk are fully covered with vector control interventions would minimize the chance for transmission to occur, but such a strategy may be prohibitively expensive.

Distance to the nearest imported case proved to be one of the most important variables for prediction of transmission risk in Swaziland, second only to the distance to the highest NDWI locations in improving model accuracy during the high season. This result indicates that imported cases from endemic neighbours are playing an important role in sparking transmission during the months of the year with highest burden, and it suggests that ongoing endemic transmission may only be occurring in limited, highly focalized regions where suitability for mosquito breeding is high. Both of these conclusions are consistent with an epidemiological context in which endemic transmission has been interrupted in the great majority of the country, and where the majority of malaria transmission might cease to occur if importation could be substantially reduced. Over 2011, there were 191 case patients with no travel history to endemic regions compared to 170 imported cases, giving a ratio of just over one local case per imported case. Such a result would be expected if R_C_, the reproductive number under control, averaged approximately 0.5 [15].

The apparent importance of imported cases for driving high-season transmission in Swaziland today also raises interesting questions about the causes of the observed seasonality in disease. Increases in local transmission occurred following the peak rainy season, and rainfall was found to be an important predictor of risk, particularly during the low season months. However, the peak of the rains also coincided with a peak in imported malaria cases following the return of travellers from endemic areas after the holiday season. Although the relative contribution of these two factors is not yet clear, they suggest the importance of a dual strategy that focuses on reducing importation while ensuring that transmission potential in high risk areas is minimised. The multivariate mixed model predicting whether or not an imported case will lead to local transmission indicates that onward transmission risk may be predictable on the basis of factors including temperature, elevation, wetness and vector control. This result suggests an evidence-based mechanism for prioritizing responses in highest risk regions.

Prediction of areas of risk during the low season produced a weaker fitting model than for the high season. In part, this result may be attributed to the fact that only 44 case patients with no travel history to endemic areas were identified during this period. As more surveillance data become available from future years, improved prediction may become possible. Nevertheless, the low season map proved useful in prospectively predicting areas at risk of local transmission in 2012 (Figure 5B). Interventions may have the greatest impact when implemented during the low transmission season [55,56], and these maps may provide a useful means for targeting those interventions. Regions at highest predicted risk were roughly consistent between the high and low season map, supporting the theory that malaria transmission in the high season may spread from hotspots that remain during the low season [6]. Understanding whether these higher-risk regions remain consistent from year to year will require further investigation.

Vector control interventions were not found to be important determinants of model accuracy. Coverage with nets and IRS was found to be higher in areas where locally acquired cases were identified, suggesting that these interventions are appropriately targeted to high-risk areas. The models generated here likely reflect a mixed effect where vector control implemented early in the time period has a negative effect on subsequent transmission, but vector control implemented later is targeted to areas where cases have recently been observed. These two effects may cancel out the observed impact of vector control in these models. Making maps with greater temporal resolution - risk over a month, for example - may better capture the effects of these interventions.

This investigation has several important limitations. Only a single usable Landsat image was identified within a similar timeframe as the surveillance data in this analysis. Temporally linked imagery for each season would improve prediction and comparison across seasons. The planned launch of the Landsat Data Continuity Mission [57] in 2013 should provide new images useful for this purpose, and the availability of processed and composited imagery through the Google Earth Engine [58] will also improve access. Similarly, high-resolution rainfall data were not available at appropriate temporal resolution. Few cases were identified during the low season, producing too small a sample size for reliable prediction. Future surveillance data may be combined across seasons to overcome this limitation. Hotspot identification using clinical malaria may be limited by the fact that higher immunity in hotspots may actually reduce development of symptoms in these higher transmission areas [6]. Nevertheless, in Swaziland, transmission appears to be so low that it is likely that this problem is minimized. It is likely that the location at which cases were investigated is not always the location at which they were infected, which would introduce error into the model. Selection of the background points was performed proportionately to population density to ensure comparability, but if only a subset of the population tended to seek care at health facilities (for example, those living nearest to clinics), these background points may differ in important ways from the locations of identified cases. Finally, not all confirmed cases were investigated by surveillance workers, and it is likely that not all malaria cases were identified by the passive surveillance system. As the system improves in detecting all malaria cases, these sorts of analyses will become more accurate.

As scale-up of vector control and effective treatment continues, other countries will join Swaziland in reducing malaria to the point where identification and elimination of the final foci of endemic transmission and prevention of onward transmission from imported cases become the goals of anti-malarial efforts. Once malaria incidence has declined to the point that geolocation of case households is operationally feasible, generation of case-based risk maps at high spatial resolution will support control programmes in targeting elimination interventions. Integrating mapping approaches into user-friendly, rapidly updateable tools [59], potentially linked to dynamic transmission models, will provide strategic, evidence-based guidance for adaptive management of malaria programmes. Efforts to create user-friendly tools based on the models generated here are underway to aid Swaziland's malaria program in rapidly updating risk maps as new data become available. This sort of case-based mapping will help ensure that the impact of limited resources is maximised to achieve and maintain malaria elimination.

## Abbreviations

AUC: Area under the curve; ETM: Landsat Enhanced Thematic Mapper; GIS: Geographic information system; IRS: Indoor residual spraying; NDVI: Normalized difference vegetation index; NDWI: Modified normalized difference water index; NMCP: National Malaria Control Programme; ROC: Receiver operator characteristic; SAGA: System for Automated Geoscientific Analyses; SMS: Short message service; TWI: Topographic wetness index; WHO: World Health Organization

## Competing interests

The authors declare that they have no competing interests.

## Authors' contributions

JMC and AJT conceived of this study. SD and SK oversaw data collection and contributed to its analysis and interpretation. JMC, SD, JMN, and AJT performed the statistical analysis and mapping. All authors contributed to interpretation of results. JC drafted the manuscript, and all authors read and approved the final manuscript.
